# Exploring the shift in newborn care practices among mothers and grandmothers in rural Odisha, India — a qualitative study

**DOI:** 10.1186/s12887-024-04916-7

**Published:** 2024-07-05

**Authors:** Livson Thomas, Sumitha Arun, Deepak Thomas Varughese, Christ Kamalini Soreng, B. Prativa Manjari, Prabhati S. Khosla, Dikhita K. Pani

**Affiliations:** 1 Christian Hospital Bissamcuttack, Rayagada District, Odisha, 765019 India; 2 Believers Church Medical College Hospital, St.Thomas Nagar, Kuttapuzha P.O, Thiruvalla, Kerala, 689103 India; 3 Department of Community Medicine, Believers Church Medical College Hospital, Kerala, India

**Keywords:** Newborn, Newborn care practices, Rural community, Accredited social health activist (ASHA), Traditional practices, India

## Abstract

**Background:**

This study delves into newborn care and infant-feeding practices in rural Odisha, specifically focusing on the rural KBK + region of Odisha (Kalahandi-Bolangir-Koraput region), inhabited predominantly by Schedule Tribes and Schedule Castes individuals. There has been an improvement in the health indicators in these areas in recent times. In the background of improved health indicators in these areas, this research explores the current and changing newborn care practices and attempts to gain insight into people’s perceptions of the factors that brought about the changes.

**Methods:**

This qualitative study was conducted between February and July 2023 at Christian Hospital, Bissamcuttack in Odisha. The methodology involved focused group discussions with mothers and grandmothers.

**Results:**

Analysis revealed healthy practices like exclusive breastfeeding till six months of age, appropriate timing of the first bath, and prompt healthcare-seeking behavior for minor illnesses among the mothers. The use of cow ghee or breast milk in a baby’s eyes, the application of ash on the umbilical cord, and the use of herbal medicines for minor illnesses were practiced more by the grandmothers in the past and were not as popular among the mothers. It is noteworthy that the cultural practices to ward off the ‘evil eye’ were practiced by both mothers and grandmothers alike. Despite the influence of traditional cultural practices on the beliefs and norms of the community, the study identified a shift in health-seeking behavior, with increased reliance on healthcare providers and safe healthcare practices. The study identifies the pivotal role of Accredited Social Health Activists (ASHAs) as a bridge between the rural communities and the healthcare system.

**Conclusions:**

This research provides valuable insights for healthcare providers aiming to enhance community-centric safe newborn care practices in rural settings. The emphasis is on the importance of understanding the current and changing local practices. This would help the healthcare providers to encourage healthy practices while eliminating the harmful practices related to newborn care through community workers like ASHA and Anganwadi workers.

**Supplementary Information:**

The online version contains supplementary material available at 10.1186/s12887-024-04916-7.

## Background

Newborn care practices in rural India are known to be influenced by cultural beliefs. They are guided by sociocultural factors rather than evidence-based medicine. A study from Nabrangpur district (one of the 8 districts collectively known as KBK + districts) in Odisha reported practices like early bathing, soaking the navel area with warm oil to dry the umbilical cord early, and blowing into the newborn’s ears to cure illnesses [1]. Ahuja et al. from rural Punjab described practices like giving honey to newborns to relieve cough, massaging with mustard oil, and rubbing the baby with asafetida for minor illnesses [2].

Some of these practices are not in alignment with the recommended standards of care. World Health Organization (WHO) recommends dry umbilical cord care as the ideal form of cord care, however in settings where harmful traditional substances (e.g. applying animal dung to the cord) are prevalent, they recommend the use of Chlorhexidine on the umbilical stump [3].

In recent years, there has been documented improvement in health indicators in rural India. In rural Odisha, the Infant Mortality Rate (IMR) reduced from 48/1000 live births in 2015 to 37/1000 live births in 2020 as per the Sample Registration System- Statistical Report 2020 [4]. The decreasing trend in IMR in these areas may be due to an improvement in the healthcare delivery systems and healthcare-seeking practices. This qualitative study was conducted among mothers and grandmothers (GMs) from rural districts of Odisha to gain insight into the current neonatal care practices and understand the people’s perception of the changes in newborn care practices.

## Methodology

### Study setting

The research study was conducted from February 1, 2023, to July 30, 2023, at Christian Hospital, Bissamcuttack (CHB), situated in the Bissamcuttack block of Rayagada district in Odisha. CHB is a 200-bed secondary care facility that provides services across various general specialties. The Obstetric department of the hospital serves pregnant women from the KBK + districts and handles approximately 4000 deliveries annually. The KBK + districts is a collection of 8 districts namely Kalahandi, Bolangir, Koraput, Nabarangpur, Nuapada, Rayagada, Malkangiri, and Sonepur in rural Odisha. The KBK + is one of the most backward areas in the country and has received several special financial aid for development from the central and state government including the Special Financial Assistance of the State of Odisha Act, 2020 [5]. The majority of the pregnant women seeking care at CHB are residents of the KBK + districts and predominantly belong to Scheduled Tribes and Scheduled Castes.

### Study participants

The participants for the study were mothers and grandmothers who were chosen through purposive sampling. Only those hailing from the KBK + districts were eligible for the study. The grandmothers of the newborns admitted to the maternity ward at CHB were included.

Multipara mothers with atleast one live child were selected from the CHB maternity ward on the second postpartum day. The participants were chosen from the hospital as they would be from different districts and the discussion would yield varied answers. However, as these are neighboring districts, the authors believed that the practices may not vary drastically among the villagers of different districts and the group would be fairly homogenous.

### Data collection tool

A qualitative approach was employed for data collection in this study. Focused Group Discussions (FGD) were used as the primary method for gathering data as the authors felt that the women in this region felt more comfortable sharing in a group setting than in face-to-face interviews. The FGDs were held at the hospital prior to discharge. The sessions were based on an interview guide designed for both mothers and grandmothers (Supplemental Table 1). Five refused to participate as they did not have anyone else to take care of the baby or were unwilling.

We obtained poor and reserved responses during one-to-one interviews with three mothers. Hence, a pilot study was conducted with two FGDs, one for mothers and the other for grandmothers. The first FGD included 12 mothers and revealed valuable insights, but challenges in participant opportunities emerged, leading to prolonged sessions. Subsequently, a focused FGD with six grandmothers was organized. This FGD provided every participant ample opportunity to share thoughts with effective interviewer control. Post-pilot, the interview questions were modified for clarity and understanding.

Basic demographic variables including age, education, ethnicity, district, yearly income, and obstetric score were collected from mothers, while grandmothers provided information on the number of grandchildren instead of the Obstetric score. The questions focused on various aspects of newborn care practices such as umbilical cord care, bathing, breastfeeding, eye care, management of jaundice, response to illnesses during the neonatal period, beliefs in the evil eye, and an exploration of the perceived changes in newborn care practices and the factors that may have caused the changes. The mothers discussed the newborn care practices they adopted for their previous children while the GMs talked about care practices followed during their time as mothers. 5 of the researchers were involved in the FGDs. Each FGD was conducted with the principal investigator (LT) as the lead interviewer along with two other interviewers. Four of them identify as female(CKS, BPM, PSK and DKP) and one as male(LT) and four of them were fluent in the vernacular language and had experience in maternal and child health nursing. The mothers and grandmothers were seated comfortably in a well-ventilated classroom within the maternity ward at CHB with provisions for drinking water during the FGDs. As the sessions would last for 45 min, we made provisions for them to attend to their baby if required, during the interview. The interviewers introduced themselves and made the participants aware of their work experience in the field of Maternity Nursing. Each discussion lasted 45 to 60 min and was conducted in the local vernacular language. The sessions were audio-recorded, and written notes were made during the session. At the end of each question in the FGD, the written notes were verified with the participants. Each FGD had a homogeneous group of 6 participants.

### Thematic analysis

The audio recordings were transcribed separately by two investigators fluent in the native language. These transcripts were then cross-referenced with the written notes to ensure data accuracy. Microsoft Excel was used to enter the data which was then coded independently by two investigators, LT and DV. An inductive method of thematic analysis was used and no preexisting coding frameworks were used. Following the generation of the initial codes, similar codes were collated and patterns were identified to develop themes (Supplemental Table 2). LT and DV then compared and discussed their coding. Data saturation was achieved when no further perspectives were emerging after 6 FGDs (3 FGDs for mothers and 3 FGDs for grandmothers). The key themes were reviewed, revised, and finalized by LT, DV, and SA by consensus. Exemplary participant quotes were noted where appropriate.

Descriptive statistics for continuous variables were expressed as median and interquartile range. Categorical variables were expressed as frequency and percentage. Data was analyzed using R software version 4.3.2.

### Ethical considerations

This study was approved by the Institutional Ethical Board at Believers Church Medical College Hospital (IEC/2023/05/336) and the Medical Superintendent of Christian Hospital, Bissamcuttack, before commencing the study. Participants were provided with a comprehensive explanation of the study’s objectives and methodology, and written informed consent was obtained before initiating the focused group discussions and audio recordings.

The Consolidated Criteria for Reporting Qualitative Research (COREQ) were followed throughout the process of data collection and reporting.

## Results

The results are based on 6 FGDs with a total of 36 participants (3 FGDs for mothers and 3 FGDs for grandmothers).

### Participants

The study included eighteen mothers and eighteen grandmothers. Among the grandmothers, the median age was 47.5 years (IQR 42.8–50). Eight grandmothers were from Rayagada district, six were from Kalahandi, and two were from Bolangir. The median age of the mothers was 25 years, and half of them were from the Rayagada district and eight were from Kalahandi. A majority of the grandmothers (15/18) had never attended school, and the highest level of education achieved among them was 12th standard. The majority of the mothers (11/18) had secondary education or above. The median annual income for each household was Rs.36,000 among grandmothers and Rs.100,000 among mothers (Table 1).

**Table 1:** Demographics of participants

Characteristic	*N* = 18(Grandmothers)	*N* = 18(Mothers)
Age, Median (IQR)	47.5 (42.8–50.0)	25.0 (23.3–31.5)
District, n		
Bolangir	2	-
Gajapati	1	1
Kalahandi	6	8
Nabarangpur	1	-
Rayagada	8	9
Number of grandchildren, Median (IQR)	2.00 (2.00–4.00)	2.00 (2.00–4.00)
Years of Education, n		
Did not go to school	15	5
Primary#	0	1
Middle	2	1
Secondary	0	6
Higher Secondary	1	3
Graduate	0	2
Annual income, Rs, Median (IQR)	36,000 (31,500–49,500)	100,000 (67,500–120,000)

*Categorical variables are mentioned as frequency (n)

#Education is classified as Primary (Class 1–5), Middle (Class 6–8), Secondary (Class 9–10) and Higher Secondary (Class 11–12)

### Use of topical agents on the umbilical cord

The practice of using topical agents on the umbilical cord was described by both mothers and grandmothers. The discussion with mothers revealed a blend of traditional approaches like sesame oil, and modern approaches like ointments provided by doctors, Chloramphenicol eye drops, Gentian Violet, and Nebasulf (Neomycin Bacitracin powder). These ointments were often supplied by the ASHA workers. Few mothers reported heating their hands with steam and applying them to the umbilical cord area with the belief that heat improves blood flow.

On the other hand, turmeric mixed with castor oil was more popular among the grandmothers. They believed that turmeric had properties that prevented infection. Some GM also infused garlic in the oil. The use of ashes such as ash from the jute rope used in cots( “*Khato Daudi*”) and coir from coconut was reported by grandmothers. They felt that garlic and ash would help the cord dry faster. However, many mothers disagreed with the use of ash and felt it should no longer be practiced, as it could cause infection in the babies.

### The traditional and modern influences on the first bath

The insightful discussion with grandmothers revealed that in the past, they would give baths to their newborns on the first day of life, meticulously removing blood from the baby’s skin to prevent infection. During the first bath, they also gave vigorous massages with turmeric-infused oils to protect the baby, as they believed the baby had become dirty during the delivery process due to blood and amniotic fluid. They also cleaned the baby’s mouth with a cloth dipped in turmeric paste to keep all germs away from the baby’s mouth. Most of the grandmothers also mentioned applying castor oil after the bath. It was warmed in the hands and applied to the baby to help the baby sleep better and to the baby’s skin soft.

On the other hand, most of the mothers bathed their newborns after discharge from hospitals, which was usually 3 days after birth. Few mothers delayed giving the first bath to the baby till the umbilical cord fell. The mothers used contemporary elements such as bathing soap, baby shampoos, and baby powders. A significant number of mothers also applied turmeric paste during the baths as they believed it had antiseptic properties and some mentioned the use of rose water and *besan* (gram flour) as they believed it would make the baby’s skin fair and glow. The mothers did not give vigorous massages as they felt it could cause harm to the babies (Table 2). Through this discussion on bathing practices, we observe a convergence of tradition and modernity in the care given to newborns, highlighting a delicate balance of traditional methods and contemporary adaptations.

**Table 2:** Emerging themes and sub-themes from the focus group discussions

Theme	Sub Theme	Codes
Umbilical cord care practices	Topical oils are used by mothers and grandmothers	Sesame oil, castor oil, warming mother’s hands on steam and placing it on the baby.
	Other topicals used mostly by mothers	Chloramphenicol eye drops, ointment prescribed by doctor, Neosporin ointment, Gentian Violet, and Nebasulf.
	Less use of burning materials by mothers	Ash of jute rope and ash of coir from coconut were more common among grandmothers.
Bath-related practices	Normal timing	Mothers gave the first bath on the day of discharge (2nd or 3rd day).
	Delayed bathing by some mothers	The first bath after the cord falls
	Early bathing by grandmothers	On the same day of birth. Vigorous massage to baby to remove blood and dirt
	Application of substances by mothers(traditional and modern influence)	Soap, sesame oil, turmeric
	Traditional topicals used by grandmothers	Turmeric and castor oil. Cleaning of mouth with cloth dipped in turmeric, warm hands over castor oil.
Breastfeeding	Correct duration of exclusive breast feeds among mothers	Exclusive breastfeeding for 6 months.
	Prolonged breastfeeding among mothers	Breastfeeds are given till 3 years. One mother till 7 years.
	Prolonged exclusive breastfeeding and prolonged duration by grandmothers	Exclusive breastfeeds were given till 8 months or more and breastfeeding is stopped only at 3 or 4 years.
Eye care practices	Routine care by mothers	Clean with water or wipe with a cloth during bath.”
	Immediate health seeking among mothers	Do not put anything. Take to hospital.”
	Other substances used mostly by grandmothers	Breast milk, kajal, oil, cow ghee.
Yellowish discoloration	Health-seeking behavior of mothers	“Sunlight and take to the hospital”- mothers
	Desi Medicine used more by grandmothers	“Cheramuli” (herbal medicine)
Minor illnesses	Health-seeking behaviour more in mothers	Take to hospital, Take to hospital with ‘ASHA didi’s’ help
	Other sources	Local pharmacy
	Local remedies(more among grandmothers)	Bhang pata (Cannabis leaf) & Korela pata (Bitter-gourd leaf)
Evil eye practices followed by mothers and grandmothers	Related to mother	Broom in hair, expressing breast milk, and throwing it outside the house.
	Related to baby	Kala tikka, dung on the baby’s forehead, mud on the baby’s forehead, bezoar (Gorochana)
	Other practices	Prayers (aarti) using salt, chilies, and mustard seeds
Perception of factors bringing about change in newborn care practices	Health-seeking behavior	Health education classed by ANM. Easy access to health care through ASHA workers and Anganwadi workers
	Related to mother	Social media and easy access to information.

### Exclusive breast feeding

Across generations, breastfeeding emerged as a timeless bond between mother and child. Grandmothers fondly recount their commitment to exclusive breastfeeding for a minimum of six months, with some of them continuing exclusive breastfeeding until their infants reached 1 year. A considerable number of grandmothers continued breastfeeding until their children reached 3 years of age, reflecting a deep-rooted belief in the benefits of breast milk.

The mothers also uphold the tradition of exclusive breastfeeding for the first six months, with many continuing to breastfeed until their children are two or three years old. However, none of the mothers continued prolonged exclusive breastfeeding beyond the first six months. Notably, the use of infant milk substitutes or baby formula appears to be a rare occurrence, with only one mother reporting to have utilized these alternatives.

During the FGD, one grandmother shared her experience of breastfeeding for five years while another mother recounted breastfeeding her baby for seven years. Additionally, one grandmother shyly smiled as she mentioned discontinuing breastfeeding upon becoming pregnant again, with a gap of two to three years between each pregnancy. This narrative highlights the enduring commitment to nurture through breastfeeding, both in the past and present.

### Practices related to eye care

The discussions on eye care practices revealed that mothers commonly use water and a clean cloth to cleanse their baby’s eyes. Some of the mothers used breast milk to cleanse the eyes of germs. Almost all mothers and grandmothers said they often apply *“Kajal”* (Kohl) to the baby’s eye after a bath as it protects the eye and wards off evil spirits. However, in the case of eye discharge, mothers stressed the importance of seeking medical attention from nearby healthcare providers rather than relying on traditional remedies. Notably, a 28-year-old mother from Bolangir expressed a preference for hospital treatment over traditional practices like using breast milk for addressing eye issues.

“If there’s a problem with my child’s eyes, I would take them to the hospital instead of following traditional practices like using oil in eyes”.

In contrast, grandmothers favored and used traditional remedies such as breast milk and cow ghee, believing in cow ghee’s potential to improve eyesight. This discussion underscores the diverse perspectives on newborn eye care, highlighting the variety of approaches and beliefs surrounding this topic.

### Yellowish discoloration of skin

A few mothers preferred to expose their newborns to sunlight when their baby had yellowish discoloration of the skin and opted for hospital care only when it did not subside with sunlight. Notably one mother expressed her willingness to expose her baby to sunlight for 2 weeks before considering hospital care if the condition persisted. However, most mothers said they would take their baby to the hospital immediately if they noticed any yellowish discoloration. Most of the mothers were doubtful about using herbal remedies like “Cheramuli”. One mother who was 33 years old said “My first baby died from jaundice because the elders in the village said there was no need for a hospital visit. They said the baby would be fine with sunlight and turmeric. Now, I will take my baby to the hospital for any problem”.

In contrast, grandmothers referred to a wide range of practices, with some using traditional herbal remedies called “Cheramuli” while others exposed their babies to sunlight and waited at least a week before seeking medical treatment.

### Treatment for minor illnesses

Abdominal colic, eye discharge, rashes on skin, nasal block, and cough are the commonly encountered minor illnesses in the newborn period.

The grandmothers used herbal medicines including formulations with cannabis leaf and bitter gourd leaf and other locally available remedies called ‘desi medicines’. The senior village members would discourage them from going to a doctor or a clinic.

“During our time we used to give desi medicines first, then if the baby didn’t become okay, we used to take the baby to a doctor or hospital.” − 40-year-old grandmother from Kalahandi district.

During the discussion, a 45-year-old grandmother from Bolangir district said “We used to have “Bhang pata” (Cannabis leaf) and “Korela pata” (Bitter gourd leaf) - through breast milk, it will go to baby, and if the baby doesn’t become ok, we take to a nearby doctor”.

All of the mothers said that they had not used and that they would not use herbal or desi medicines to treat any illness in their newborns. Instead, they would go to a hospital for any illness related to the baby. The first stop for help (apart from family/neighbors) seemed to be the Accredited Social Health Activists (ASHAs) or the local pharmacy. Only one of the mothers said she would go to the hospital only if the baby was very sick. This thought process appeared to be an exception rather than a rule.

### Practices to ward off ‘Evil Eye’

The belief in the ‘evil eye’ is deeply set within the cultural fabric of India. To understand this, participants were asked about practices that help protect against it. Interestingly, there were no discernible differences in the practices mentioned by both mothers and grandmothers. The most prevalent practice identified was the application of “kala tikka” (black dot) on the baby’s face and forehead, typically made using kohl. Additionally, many mothers and grandmothers spoke of the custom of carrying a piece of broom in their hair to ward off evil spirits and the evil eye. Furthermore, two mothers described a ritual they followed upon returning home from outings, involving expressing breast milk, throwing it on the ground, and spitting on it, as they believed this act prevented evil spirits from harming their babies. Other remedies to ward off the evil eye included applying a tikka made from cow dung or using a bezoar, also known as ‘Gorochana’(Bezoar). Additionally, during the focus group discussions, both groups mentioned the ritual of throwing salt, along with seven mustard seeds or seven chillies, into the fire as another method to counteract the evil eye. These discussions shed light on the various cultural practices employed to protect newborn babies against the evil eye, reflecting the deeply rooted beliefs and traditions within our society.

### Perception about the factors influencing the changes in newborn care practices

Both mothers and grandmothers seemed to agree that changes have occurred in newborn care practices. We explored to understand their perception of the factors contributing to the changes in traditional newborn care practices.

The majority of the mothers and grandmothers mentioned that ASHA, Anganwadi workers and Auxiliary Nurse Midwife (ANMs) do home visits and give health education to pregnant women and mothers on the importance of antenatal check-ups, institutional deliveries, exclusive breastfeeding, and immunization. Two of the mothers mentioned that ASHA workers conducted group classes and role-play-based teaching in their villages. Most of the grandmothers mentioned that now there is easier access to information on newborn care through mobile phones and televisions.

## Discussion

Traditional newborn care practices are often handed down through generations. A high prevalence of such practices has been reported in various rural communities of India [6, 7]. There has been a significant improvement in health indicators like IMR in these communities as per the Sample Registration system 2020 [4]. In this context, our study explored the current and changing practices related to newborn care and infant feeding in a rural community of Odisha. Focus group discussions with mothers and grandmothers were employed as FGDs capture perceptions and beliefs that may not be easily understood using quantitative tools.

The World Health Organisation (WHO) recommends dry umbilical cord care [8] for institutional deliveries and topical Chlorhexidine for areas with high neonatal mortality and in home deliveries. Despite these clear recommendations, the application of various substances on the umbilical cord stump has been a common practice in rural areas. A study by Keserton et al. in rural Karnataka noted that turmeric, castor oil, and powder were commonly applied on the umbilical cord [9]. In our study, we found that the use of ash from burning different substances was more common among grandmothers. On the other hand, the use of oils, steam, and topical ointments from the local pharmacy or hospital was reported by the mothers. Gentian violet was the most common topical agent and was used under the guidance of the ASHA worker. The use of gentian violet is not a harmful practice but there is more evidence on the benefits of Chlorhexidine. Gentian violet was the most commonly used antiseptic until the community trials using Chlorhexidine in areas with high neonatal infections and mortality showed reduced neonatal deaths [10].

There is a palpable shift from the practice of using ash in the past, to using medicated creams as prescribed by health care providers. Some of the participants (mostly mothers) were aware of the possible harm related to the use of ash on the umbilical cord.

It is understood from this study, that in the past, the first bath used to be given on the first day of birth. It involved the vigorous massaging of the baby. However, to reduce the risk of hypothermia, the first bath of the baby is recommended after the first 24 h of life [9]. The current practice as described by the mothers was more aligned with these recommendations. It included a first bath on day 2 or 3 of life, with sesame oil or turmeric, and soap.

WHO recommends exclusive BF till six months of age, complementary feeding from 6 months onwards, and continued BF until two years of age [11]. The national exclusive BF rate as per the National Family Health Survey (NFHS) 2019–2021 was 63.7% and the state average was 72.9% [12]. As compared to the NFHS data, our study participants (both mothers and grandmothers) exhibited a higher rate of exclusive BF. Even though some of the grandmothers continued exclusive BF beyond six months, most of the mothers started appropriate complementary feeding after six months of life with continued BF. The deep-rooted belief of the community in breastfeeding is evident among all of the participants.

There were no significant differences in the practices and the viewpoints regarding evil eye practices among mothers and grandmothers. Practices included keeping a broom in the mother’s hair, kala tikka (black mark) on the baby, and even aarthi (traditional prayers) using chilly and mustard seeds. Evil eye practices are community-specific and continue to be practiced, as they are intricately embedded within the fabric of the communities [13, 14]. Some of the practices may be harmful and need to be identified and changed while respecting the ethos of the community. It is important to consistently engage with the community through health education to eliminate such harmful practices.

Previous studies reported poor healthcare-seeking behavior and low healthcare utilization in the rural communities of India [15]. It is clear from the grandmothers in the study that traditional home remedies were previously the first option for an ailment. However, most of the mothers mentioned approaching healthcare providers like ASHA workers or the hospitals directly for all health-related concerns. This study highlights the significant role played by hospitals and healthcare providers in recent times. The ‘ASHA didi’ (as she is referred to, didi means sister) appears to be an effective ‘bridge’ between the rural people and the healthcare system. Her integration into the healthcare system is one of the key points of change, mentioned by the participants. The pivotal role played by ASHA workers was discussed in previous studies where they describe the adequate coverage of Home-Based Newborn Care by ASHAs and their role in the utilization of maternity-related services [16, 17].

### Strengths and limitations of the study

Most studies from rural India have focused on the harmful traditional practices and evil eye practices in a tribal or rural community. Our study explored the newborn care practices among mothers and grandmothers which helped us understand not just the current practices, but also the practices from different periods. We also explored the people′s perception of the practices that are not followed anymore, and the factors that brought about the changes.

As we used FGDs, we had to limit the study to a few questions that would shed light on common newborn care practices. The research was conducted in a hospital setting and not in the community. The authors believed that a hospital-based study would reveal the practices in the area as the institutional deliveries are more than 90% [18]. The hospital setting provided us with ease of access and with the manpower we had, we could conduct each FGD with three interviewers. However, we acknowledge that the results may reflect the practices among people with high healthcare-seeking behavior and may not represent the subset of people who do not seek healthcare.

## Conclusion

Our study sheds light on the newborn care and infant feeding practices in a rural community in Odisha. Traditional practices persist, especially in umbilical cord care and evil eye practices. Notable shifts in healthcare-seeking behavior, with a growing reliance on healthcare providers, especially ASHA workers, ANM nurses, and Anganwadi workers are evident. These health workers have become trusted advisors in these communities and work in unison to improve healthcare utilization.

It is important to maintain a delicate balance between cultural heritage and evolving healthcare trends. This study helps us understand existing local practices and may help us develop culturally sensitive approaches to engage with the community.

### Electronic supplementary material

Below is the link to the electronic supplementary material.

Supplementary Material 1

Supplementary Material 2

## Data Availability

The datasets collected and analyzed during the current study are available from the corresponding author upon reasonable request.

## Abbreviations

KBK +: Kalahandi, Bolangir, Koraput
ASHA: Accredited Social Health Activist
WHO: World Health Organisation
IMR: Infant Mortality Rate
GM: Grandmother
CHB: Christian Hospital, Bissamcuttack
FGD: Focus Group Discussion
BF: Breastfeeding
ANM: Auxiliary Nurse Midwife
NFHS: National Family Health Survey
