# Supplementary material for: Exploring the shift in newborn care practices among mothers and grandmothers in rural Odisha, India — a qualitative study
Source: BMC Pediatr. 2024 Jul 5;24:432. doi: 10.1186/s12887-024-04916-7 (PMC11225211; doi:10.1186/s12887-024-04916-7)
Supplement: Supplementary file 1 — Supplementary Material 1 [file 12887_2024_4916_MOESM1_ESM.docx]

**SUPPLEMENTAL TABLE 1 : INTERVIEW GUIDE FOR MOTHERS**

1. How did you care for the umbilical cord of your previous children? Did you apply anything on the cord both before and after falling off the cord? If yes, what did you apply?

2. At what age did you give your child/children their first bath? How was it given and what were the topicals used during bathing?

3. Till what age did you breastfeed your child? How long was exclusive breastfeeding done and what were the complementary feeding practices you followed?

4. What were the eye care practices you followed for your child as a routine and during eye discharge if any?

5. Did your child have yellowish discoloration of skin in the first month of life? If yes, what remedial measures did you take? If not, what measures would you have taken if your child had developed yellowish discoloration?

6. Did your child have any minor illness in the first month of life? If so, what did you do or what would you have done if the baby had developed an illness?

7. What are the evil eye beliefs you practiced when your child was less than one month of age?

8. To the best of your knowledge, what are the newborn care practices that have changed or are not followed anymore among the people around you?

9. To what and to who can we attribute, the changes you have perceived (if any), in newborn care practices?

**INTERVIEW GUIDE FOR GRANDMOTHERS**

1. How did you care for the umbilical cord of your children? Did you apply anything on the cord both before and after falling off the cord? If yes, what did you apply?

2. At what age did you give your child/children their first bath? How was it given and what were the topicals used during bathing?

3. Till what age did you breastfeed your child? How long was exclusive breastfeeding done and what were the complementary feeding practices you followed?

4. What were the eye care practices you followed for your child as a routine and during eye discharge, if any?

5. Did your child have yellowish discoloration of skin in the first month of life? If yes, what remedial measures did you take? If not, what measures would you have taken if your child had developed yellowish discoloration?

6. Did your child have any minor illness in the first month of life? If so, what did you do or what would you have done if the baby had developed an illness?

7. What are the evil eye beliefs you practiced when your child was less than one month of age?

8. To the best of your knowledge, what are the newborn care practices that have changed or are not followed anymore since your time as a mother till now?

9. To what and to who can we attribute, the changes you have perceived (if any), in newborn care practices?
