# Supplementary material for: Exploring the shift in newborn care practices among mothers and grandmothers in rural Odisha, India — a qualitative study
Source: BMC Pediatr. 2024 Jul 5;24:432. doi: 10.1186/s12887-024-04916-7 (PMC11225211; doi:10.1186/s12887-024-04916-7)
Supplement: Supplementary file 2 — Supplementary Material 2 [file 12887_2024_4916_MOESM2_ESM.docx]

**SUPPLEMENTAL TABLE 2: CODING FRAMEWORK**

| **Theme** | **Sub Theme** | **Codes** |
| --- | --- | --- |
| Umbilical cord care practices | Topical oils were used by mothers and grandmothers | Sesame oil, castor oil, warming mother's hands on steam and placing it on the baby. |
|  | Other topicals used by mothers | Chloramphenicol eye drops, ointment prescribed by doctor, Neosporin ointment, Gentian Violet, and Nebasulf. |
|  | Less use of burning materials by mothers | Ash of jute rope and ash of coir from coconut were more common among grandmothers. |
| Bath-related practices | Normal timing | Mothers gave the first bath on the day of discharge (2nd or 3rd day). |
|  | Delayed bathing by some mothers | The first bath after the cord falls |
|  | Early bathing by grandmothers | On the same day of birth. Vigorous massage to baby to remove blood and dirt |
|  | Application of substances by mothers(traditional and modern influence) | Soap, sesame oil, turmeric |
|  | Traditional topicals used by grandmothers | Turmeric and castor oil. Cleaning of mouth with cloth dipped in turmeric, warm hands over castor oil. |
| Breastfeeding | Correct duration of exclusive breast feeds among mothers | Exclusive breastfeeding for 6 months. |
|  | Prolonged breastfeeding among mothers | Breastfeeds are given till 3 years. One mother till 7 years. |
|  | Prolonged exclusive breastfeeding and prolonged duration by grandmothers | Exclusive breastfeeds were given till 8 months or more and breastfeeding is stopped only at 3 or 4 years. |
| Eye care practices | Routine care by mothers | Clean with water or wipe with a cloth during bath." |
|  | Immediate health seeking among mothers | Do not put anything. Take to hospital." |
|  | Substances used mostly by grandmothers | Breast milk, kajal, oil, cow ghee. |
| Yellowish discoloration | Health-seeking behavior of mothers | "Sunlight and take to the hospital"- mothers |
|  | Desi Medicine used more by grandmothers | "Cheramuli" (herbal medicine) |
| Minor illnesses | Health-seeking behavior more in mothers | Take to hospital, Take to hospital with ‘ASHA didi's’ help |
|  | Other sources | Local pharmacy |
|  | Local remedies(grandmothers) | Bhang pata (Cannabis leaf)  & Korela pata (Bitter-gourd leaf) |
| Evil eye practices followed by mothers and grandmothers | Related to mother | Broom in hair, expressing breast milk, and throwing it outside the house. |
|  | Related to baby | Kala tikka, dung on the baby's forehead, mud on the baby's forehead, bezoar (Gorochana) |
|  | Other practices | Prayers (aarti) using salt, chilies, and mustard seeds |
| Perception of factors  bringing about change in newborn care practices | Health-seeking behavior | Health education classed by ANM. Easy access to health care through ASHA workers and Anganwadi workers |
|  | Related to mother | Social media and easy access to information. |
